# The synthesis and properties of mitochondrial targeted iron chelators

**DOI:** 10.1007/s10534-022-00383-8

**Published:** 2022-04-02

**Authors:** Agostino Cilibrizzi, Charareh Pourzand, Vincenzo Abbate, Olivier Reelfs, Laura Versari, Giuseppe Floresta, Robert Hider

**Affiliations:** 1 Institute of Pharmaceutical Science, King’s College London, London, UK; 2 Department of Pharmacy and Pharmacology, University of Bath, Bath, UK; 3 Centre for Therapeutic Innovation, University of Bath, Bath, UK

**Keywords:** Iron chelation, Mitochondria, Chelator synthesis, Iron-probe

## Abstract

Iron levels in mitochondria are critically important for the normal functioning of the organelle. Abnormal levels of iron and the associated formation of toxic oxygen radicals have been linked to a wide range of diseases and consequently it is important to be able to both monitor and control levels of the mitochondrial labile iron pool. To this end a series of iron chelators which are targeted to mitochondria have been designed. This overview describes the synthesis of some of these molecules and their application in monitoring mitochondrial labile iron pools and in selectively removing excess iron from mitochondria.

## Introduction

Iron represents a vital element required for almost all forms of life. Because of its ability to transfer electrons by shuttling between ferrous and ferric forms, it is a crucial component participating in the electron transport chain within mitochondria. In biological systems, iron can be incorporated into iron–sulfur [Fe–S] clusters and the heme molecule (Paul and Lill 2015; Ponka 1999). Both of these iron-containing structures serve as cofactors for many enzymes and are partially synthesized inside mitochondria, rendering the mitochondrion the central organelle in cellular iron metabolism. This critical dependence of mitochondria on iron renders the specific quantification of the mitochondrial labile iron pool in both normal and disease states highly desirable (Urrutia et al. 2014). Furthermore, as labile iron is redox active, its presence will contribute to the production of toxic reactive oxygen species—(ROS). Thus, in some disease states it will be an advantage to have the capability of controlling levels of mitochondrial labile iron (Reelfs et al. 2016; Sandoval-Acuna et al. 2021).

### Neurodegeneration

Neurodegenerative diseases are a heterogeneous group of disorders characterized by gradually progressive, selective loss of anatomically or physiologically related neuronal systems. Prototypical examples include Alzheimer’s disease (AD), Parkinson’s disease (PD). It is clear that mitochondrial involvement is an important common theme in both of these disease states. Mitochondria are key regulators of cell survival and death and have a central role in ageing (Lin and Beal 2006). There is strong evidence that mitochondrial dysfunction occurs early and acts causally in the disease pathogenesis. Furthermore, it is clear that many neurodegenerative diseases are associated with accumulation of iron in the central nervous system (Ke and Qian 2003) and it will be important to establish the changes in mitochondrial labile iron levels between normality and the diseased state.

Pathological levels of iron are found in association with aggregated amyloid-β protein (A β) in the extracellular space of AD patients. There is evidence that iron accumulation in affected regions of the AD brain may outstrip the ability of ferritin to safely sequester it, which would lead to pathological ROS formation through Fenton chemistry (Connor et al. 1992). Indeed, oxidative stress is a well-documented feature of AD and there is evidence to support the concept that iron contributes to oxidative stress in AD (Altamura and Muckenthaler 2009). Clearly there is an urgency to establish the labile iron levels in Alzheimer brain mitochondria.

Iron accumulation in substantia nigra has been a recognised feature of Parkinson’s disease (PD) (Dexter et al. 1987). Excessive cellular iron, through its propensity to generate ROS, has consequences for many of the well-established pathogenic mechanisms of PD. Mitochondrial dysfunction was one of the earliest features identified in the study of subcellular pathology in PD. Reduced activity of mitochondrial complex I has been reported in the brain, platelets, and skeletal muscle of patients with idiopathic PD (Krig et al. 1992). A number of chelators have been directed at the removal of iron from the brains of PD patients, the most extensively studied being deferiprone (Devos et al. 2014). A disadvantage of deferiprone is that it lacks a controlled cellular distribution.

### Cardiovascular disease

It is well established that many cardiovascular disease risk factors are associated with mitochondrial dysfunction, for instance atherosclerosis, hypercholesterolemia, diabetes, ischemia–reperfusion injury and exposure to tobacco smoke. Indeed, the iron chelator deferiprone is capable of removing iron from mitochondria and reversing the symptoms of chronic obstructive pulmonary disease in mice (Cloonan et al. 2016). The disadvantage of using deferiprone, as indicated above, is that it is not targeted to the mitochondria.

### Skin photodamage

The presence of appreciable levels of labile iron in the mitochondria of fibroblasts and keratinocytes render these cells sensitive towards oxidative damage on exposure to ultraviolet A (UVA, 320–400 nm, the oxidising component of sunlight). The harmful consequences of iron-catalysed damage exerted by UVA have been shown to play a key role in skin photoaging and photocarcinogenesis (Dissemond et al. 2003). In this regard, solar UVA is recognised as a strong membrane damaging agent promoting lipid peroxidation in subcellular organelle membranes of skin fibroblasts and keratinocytes via pathways involving singlet oxygen and labile iron (Vile and Tyrrell 1995). Furthermore, the labile iron-mediated oxidative damage caused by UVA to mitochondrial membranes has been shown to cause necrotic cell death via ATP depletion (Zhong et al. 2004; Aroun et al. 2012).

### Mitochondrial-targeted chelators

**Fig. 1:**
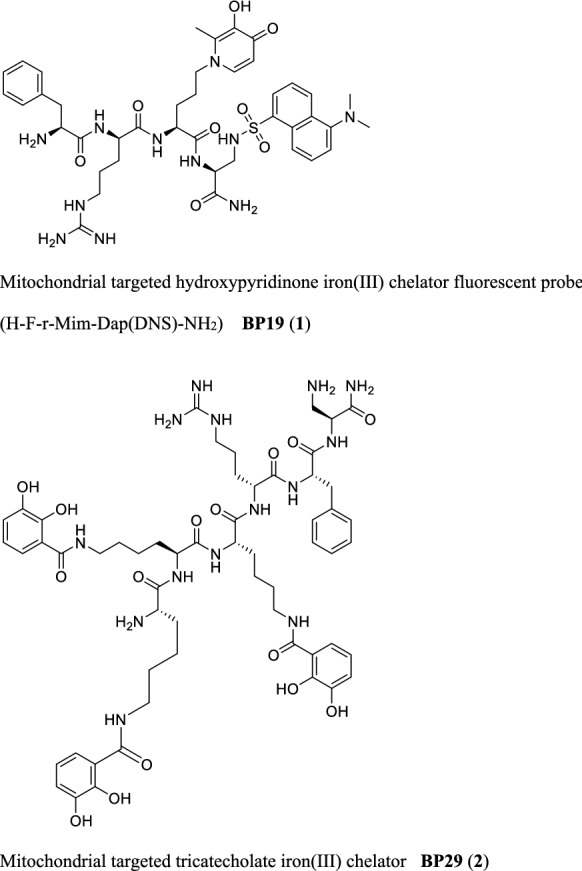
Structure of BP19 and BP29

Because of the crucial role of mitochondrial iron overload in a growing number of oxidative conditions and pathologies, there is a clear need to design mitochondrial iron probes with high specificity and sensitivity to evaluate the labile iron of the organelles (Rouault 2016; Gao et al. 2021; Mena et al. 2015). The criteria for the design of mitochondria-specific iron sensors imply that iron sensors reside exclusively in the organelles and provide a reliable quantification of mitochondrial labile iron. In this regard, we have developed a series of highly specific fluorescent mitochondria-targeted iron sensors that fulfil the above criteria as they can reach mitochondria by means of mitochondria-homing peptide sequences (Abbate et al. 2015, 2016). This successful design was initially reported by Schiller et al. (2000) and Zhao et al. (2003), whereby tetrapeptides with alternating aromatic and basic amino acids were demonstrated to penetrate cell membranes and to concentrate in mitochondria. By incorporating D-amino acids in the C-amidated peptides, resistance to peptidase attack is achieved. Although a wide range of arginine containing peptides have been reported to be able to permeate membranes and to concentrate in the mitochondria (Futaki 2006; Horton et al. 2008; Nakose et al. 2012), the SS-like peptides (Schiller and Szeto peptides) possess the advantage of being tetrapeptides (Zhao et al. 2003) and therefore they lack a pronounced secondary structure; a clear advantage for drug design. By adopting this alternating aromatic/basic residue peptide design, Horton et al (2008) and Abbate et al. (2015, 2016) have prepared fluorescent mitochondria-targeted peptides (e.g. **BP19**) which are capable of being selectively accumulated by mitochondria. The net positive charge of these peptide derivatives ensures that they are accumulated by mitochondria due to the large membrane potential of the inner mitochondrial membrane. Furthermore, by attaching an iron-chelating residue (Fig. 1), such peptides are capable of evaluating the mitochondrial labile iron pool (Abbate et al. 2016). Leading on from these findings, Reelfs et al. using hexadentate iron chelators, covalently attached to mitochondrial-targeted peptides (e.g. **BP29**, Fig. 1), have demonstrated that such molecules can selectively scavenge excess iron from mitochondria with beneficial effects (Reelfs et al. 2016).

In parallel with the above studies, Espósito et al. (Alta et al. 2017a) have reported triphenylphosphonium-deferoxamine as a candidate mitochondrial targeted iron chelator. They demonstrate that this derivatised siderophore can penetrate plasma membranes and is clearly accumulated in mitochondria. More recently, triphenyl phosphonium-deferoxamine has been demonstrated to suppress tumour growth and metastasis by scavenging mitochondrial labile iron (Sandoval-Acuña et al. 2021). However, a potential toxicity issue with the application of the triphenyl phosphonium moiety is that it can induce non-specific effects due to the accumulation of the extremely lipophilic TPP^+^ cation into membranes (Smith et al. 2003; Murphy, 2008). This issue is avoided by the utilisation of readily biodegradable peptides, such as the SS-like peptides which are reported in the present study. Significantly, Espósito et al. have also reported the application of basic/amphiphilic peptide address systems for the targeting of deferoxamine to mitochondria (Alta et al. 2017b).

Another interesting mitochondrial directed iron(II) probe is SiRhoNox-1 (mito-Ferro Green) (Hirayama et al. 2013, 2017, 2019), although its precise mode of interaction with Fe^2+^ ions still remains to be confirmed. The Fe^2+^—mediated deoxygenation of the N-oxide group on the fluorophore leads to an enhanced fluorescence. The sensitivity towards Fe^2+^ is limited to approximately 1 μM and the reduction of the N-oxide may also be facilitated by reaction with glutathione, a thiol that reaches high mM concentrations in the mitochondria. Consequently, it would appear that this probe should be subjected to more carefully controlled studies, before it can be recognised as a truly specific mitochondrial iron(II) probe.

## Results

### Synthesis of BP19 (1)

#### Solid-phase peptide synthesis (SPPS)

**Scheme 1:**
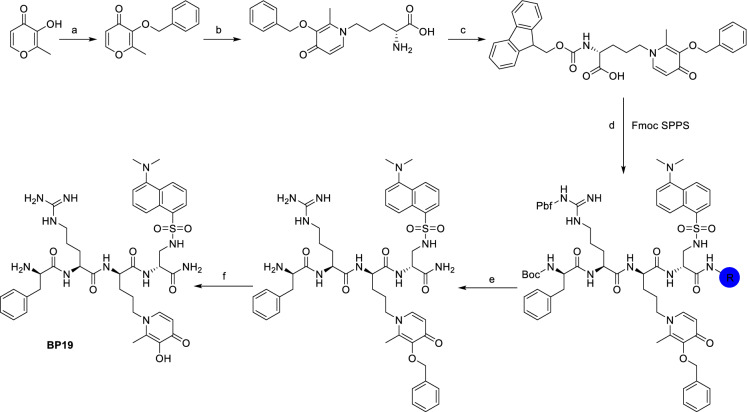
SPPS synthesis of **BP19**. Conditions: a: BnCl; b: l-ornithine, water/ethanol, reflux; c: Fmoc-O-Su, DIPEA, water/acetonitrile; d: standard Fmoc solid-phase peptide synthesis; e: TFA/TIPS/DCM 1/2/97 v/v 3 h; f: BCl_3_, DCM 3 h

Abbate et al. recently reported the synthesis of **BP19 (1)** using a solid-phase peptide synthesis (SPPS) approach (Abbate et al. 2015). SPPS is generally the first method of choice for the chemical synthesis of peptides. The derivatised mimosine analogue (Scheme 1d) was attached to a solid-phase resin as indicated in Scheme 1. A two stage deprotection (Scheme 1e and f) led to the isolation of crude **BP19** as an amorphous white solid (80% yield). Further purification was achieved by preparative high-pressure liquid chromatography (HPLC).

#### Solution synthesis

Unfortunately, although SPPS can be automated and is scalable, it suffers from a negative environmental footprint mainly due to extensive solvent use and low yields (Martin et al. 2020). In line with the modern effort of academia and industry to render peptide synthesis greener and to optimize the yields, we here report an alternative solution-based synthetic method for **BP19**.

**Fig. 2:**
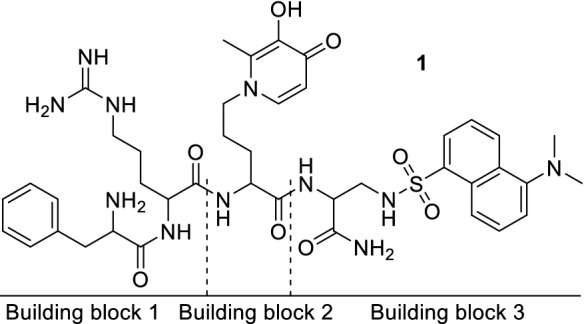
Structure of molecule **1** (**BP19**) and building blocks 1–3

**Scheme 2:**
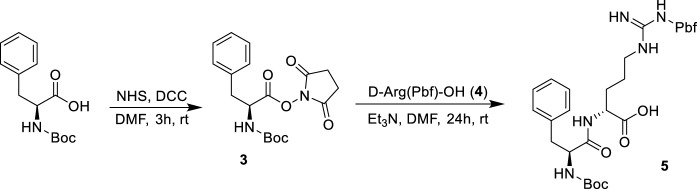
Synthesis of building block 1 (**5**)

**Scheme 3:**
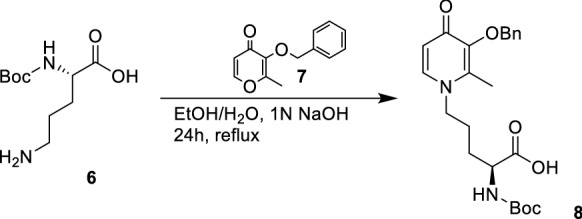
Synthesis of building block 2 (**8**)

**Scheme 4:**
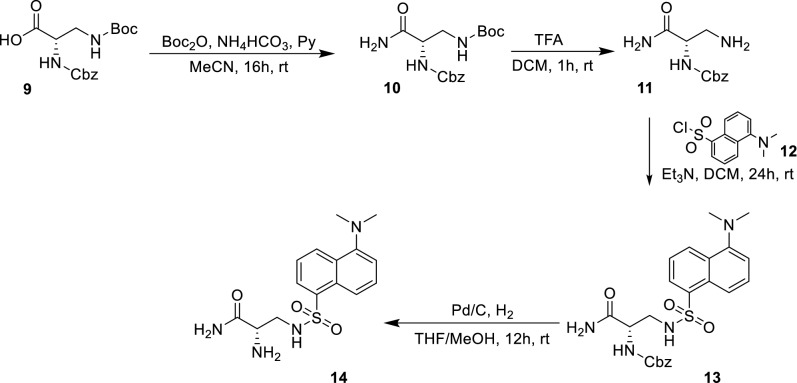
Synthesis of building block 3 (**14**)

**Scheme 5:**
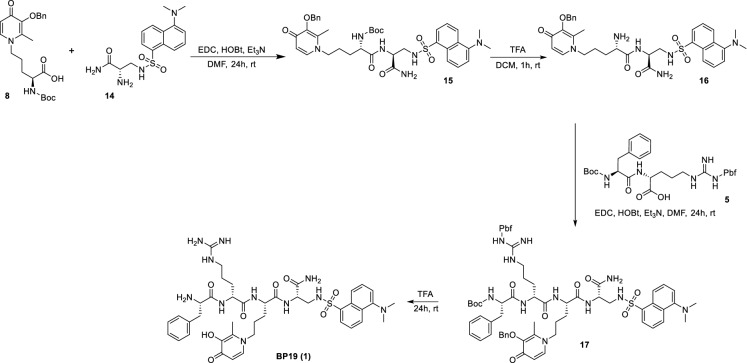
Synthesis of **BP19** (**1**) by assembling building blocks 1–3

As reported in Fig. 2, our strategy was to divide the final structure into three building blocks (BBs) choosing a convergent synthetic approach over a sequential approach in order to achieve higher yields. These three BBs were synthesized singularly as reported in Schemes 2, 3, and 4 and were then used for the final assembly (Scheme 5) of the product **BP19** (**1**).

The synthesis of BB1 (Scheme 2) was designed as a two-step reaction starting from commercially available Boc-l-Phe-OH that was first activated with DCC/NHS to produce the NHS derivative **3** and then coupled with d-Arg(Pbf)-OH (**4**) to produce the first BB (**5**). The synthesis of BB2 (Scheme 3) was achieved with one step reaction starting from Boc-l-Orn-OH (**6**) and 3-(benzyloxy)-2-methyl-4H-pyran-4-one (**7**). Molecule **7** was produced as previously reported (Cilibrizzi et al. 2018) and then reacted as classically reported with the amino group of **6** converting the pyran-4-one ring of **7** in the derivative *N*-alkylpyridones **8** (BB2). BB3 was synthesized in four stepwise reactions (Scheme 4) starting from Z-l-Dap(Boc)-OH (**9**). Molecule **9** was firstly converted into its amide derivative **10**. The reaction was initially tried with ammonia under different conditions (1. Et_3_N, ClCOOEt, THF, -5 °C to rt in 2 h and 2. EDC, HOBt, Et_3_N, DMF, rt in 24 h), but higher yields were achieved when the reaction was conducted as reported by Talukdar et al (2005) with Boc_2_O, ammonium bicarbonate and pyridine in acetonitrile at room temperature for 16 h. The resulting amide (**10**) was then deprotected at its β amino group with TFA and the deprotected compound **11** was coupled with dansyl chloride (**12**) giving compound **13**. BB3 (**14**) was finally obtained by deprotection of the amino group of **13** with 10% palladium on carbon under an atmosphere of H_2_ for 12 h.

The final assembly (Scheme 5) of **BP19** (**1**) started from the coupling of BB2 (**8**) and BB3 (**14**). The reaction was achieved using EDC/HOBt as coupling reagents to give the product **15** which was subsequently deprotected with TFA giving intermediate **16**. BB1 (**5**) was then coupled with molecule **16** with EDC/HOBt giving the protected target molecule **17**. Treatment of molecule **17** with neat TFA for 24 h facilitated the simultaneous deprotection of the labile -Boc and -Pbf protecting groups and the -Bn group on the hydroxypyridinone ring giving the final molecule **1** (**BP19**).

### Synthesis of BP29 (2)

**Scheme 6:**
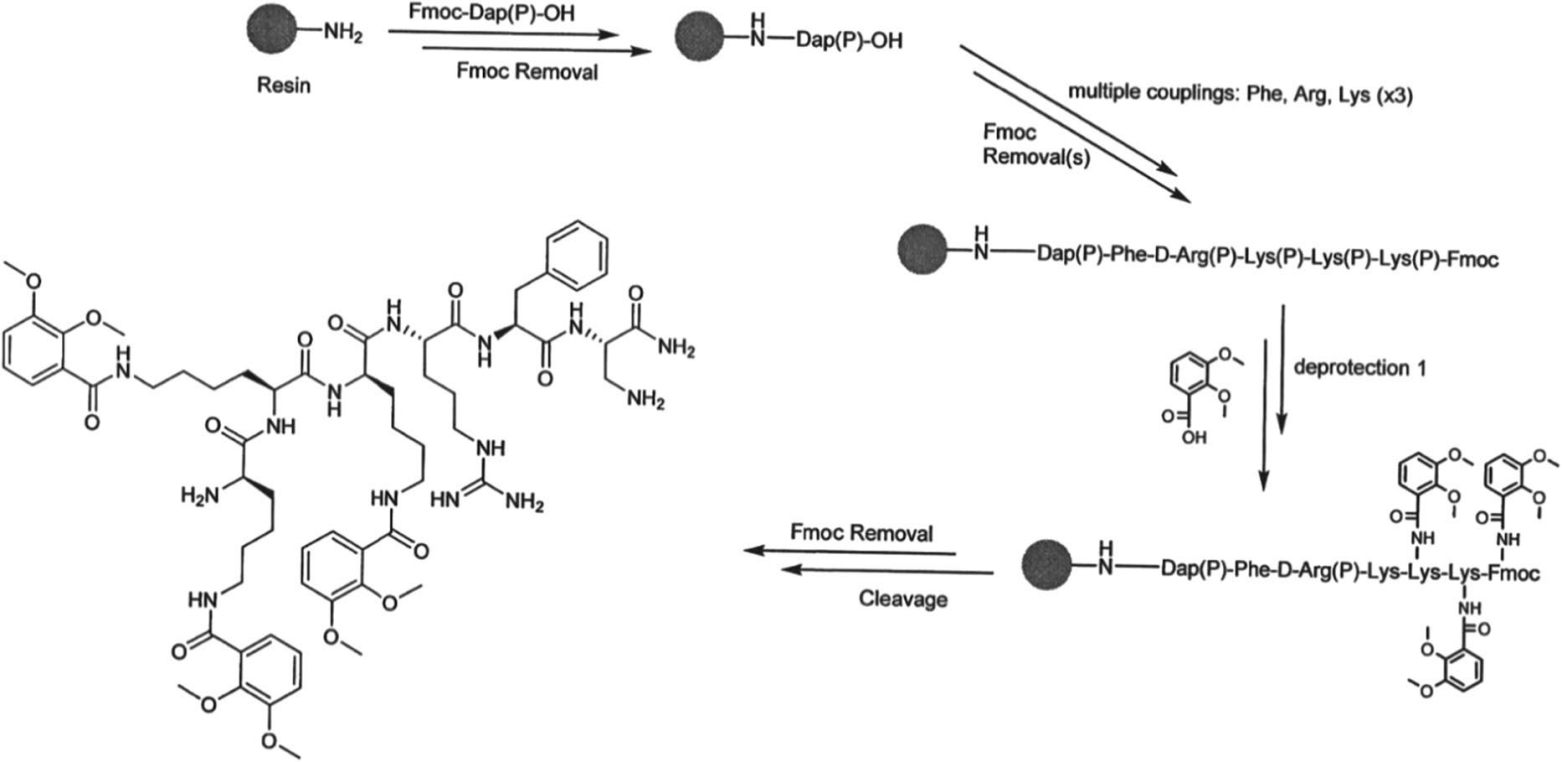
SPPS of **BP29**

**BP29** was synthesised using SPPS in similar fashion to that of **BP19** (Scheme 6). Each of the three lysine residues on the resin-bound peptide were deprotected and then conjugated to *O*-dimethyl-2,3-dihydroxybenzoic acid. Final deprotection led to the production of crude **BP29** as a white amorphous solid (80% yield). This product was purified by preparative HPLC.

### Properties of the mitochondria-targeted iron sensor BP19

**Fig. 3:**
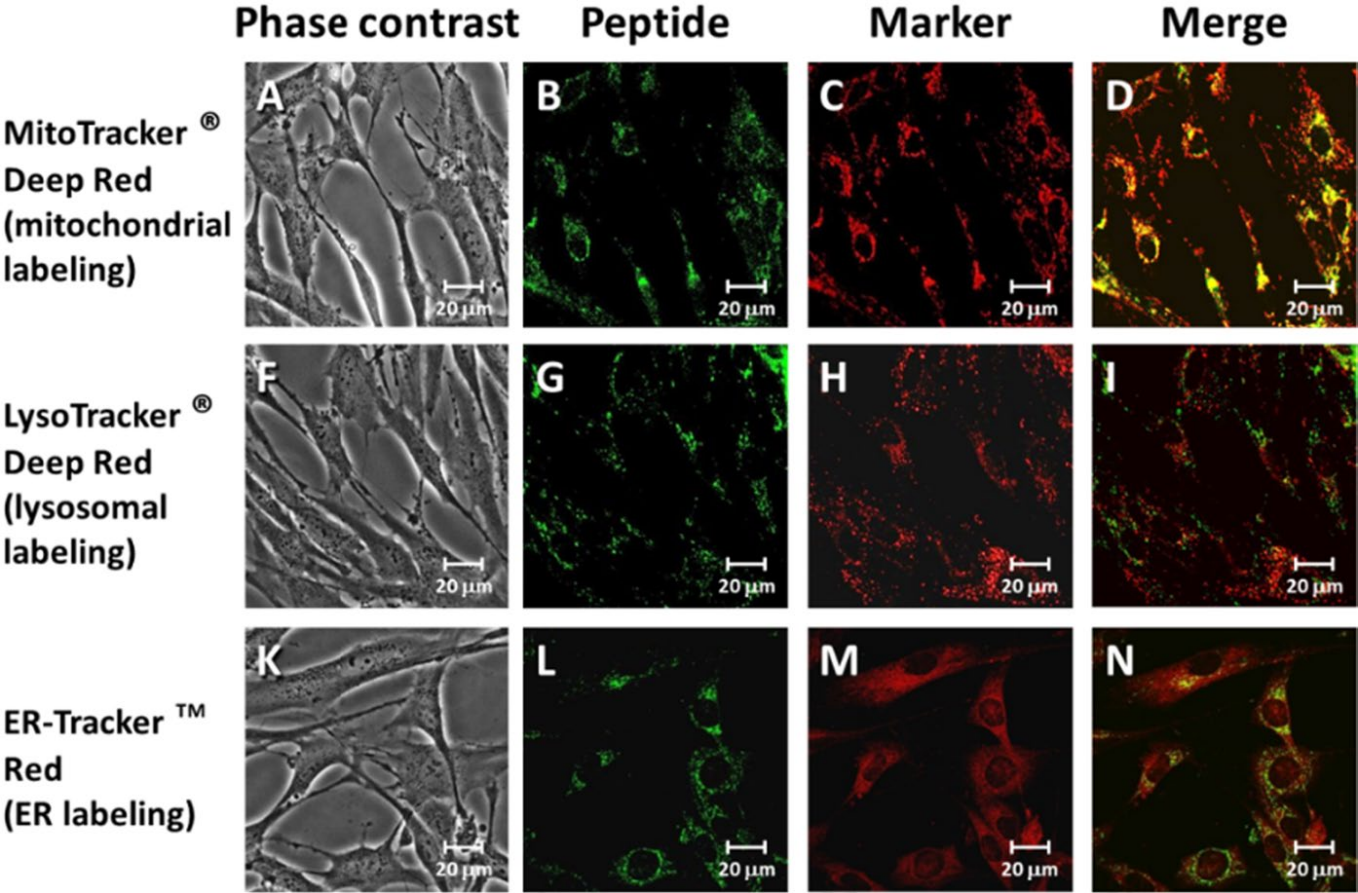
Subcellular distribution of BP19 in FEK4 cells. Representative microscopy images of subcellular localization studies of DNS(Dansyl sulphate)-labelled peptide H-F-r-Mim-Dap(DNS)-NH_2_ (**BP19**) with mitochondrial (**A**–**D**), lysosomal (**F**–**I**) and ER (**K**–**N**) compartments

**Fig. 4:**
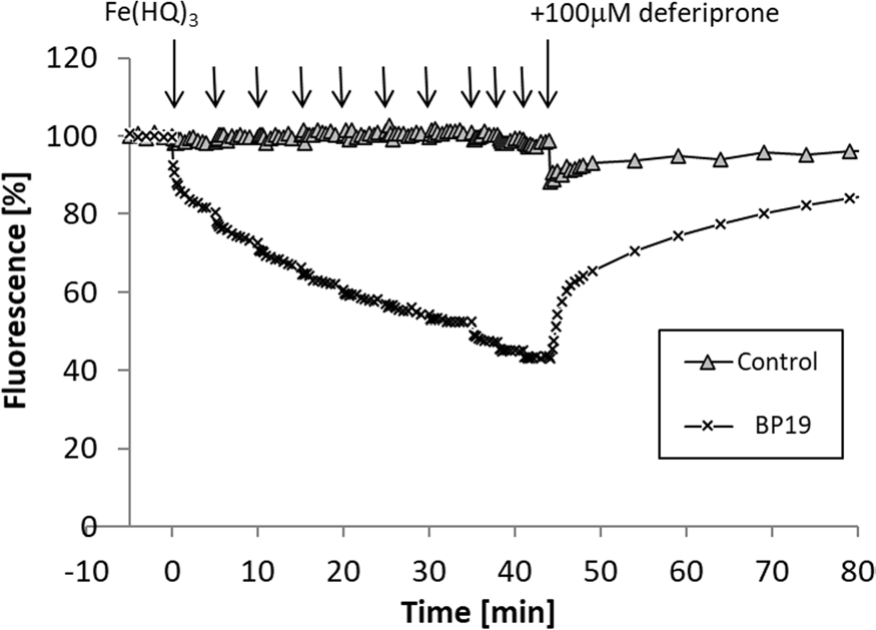
Fluorescence quenching and dequenching of BP19, FEK4 cells in response to the manipulation of cellular levels of iron. Cells incubated (or not) with peptide were loaded with iron(III) in the form of iron hydroxyquinoline complex, Fe(HQ)_3_ and then treated with a 100 μM bolus of the iron-specific chelator deferiprone. The arrows illustrate the timepoints at which additional aliquots of Fe(HQ)_3_ or deferiprone were added. A representative experiment is depicted

**BP19** was selected from a range of probes for the selective measurement of labile iron in the mitochondria. It was demonstrated that **BP19** was preferentially accumulated in the mitochondria of FEK4 cells (human primary foreskin fibroblasts) Fig. 3 (Abbate et al. 2015). Furthermore, when the cells were loaded with iron the fluorescence signal was reduced (Fig. 4) and when the added iron was chelated by deferiprone, the fluorescence was reinstated.

**Fig. 5:**
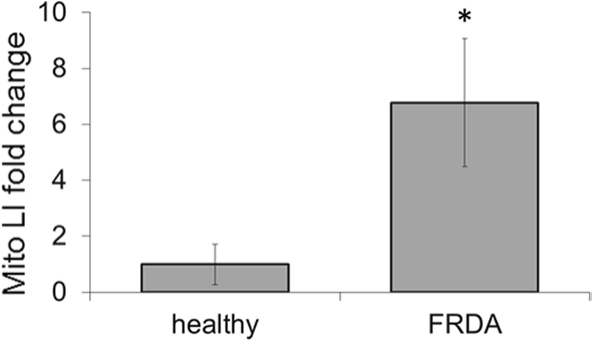
Mitochondria from FRDA fibroblasts have significantly higher levels of labile iron compared to healthy FEK4 fibroblasts. Cells were first treated (or not) with the powerful iron chelator desferrioxamine at a concentration of 100 μM and then incubated with 50 μM of BP19. Desferrioxamine pre-treatment aimed to deplete the intracellular level of labile iron (LI) and therefore allow capturing the total fluorescence of BP19 in the desferrioxamine + BP19-treated cells, as measured on a spectrofluorimeter. This total fluorescence was then compared to the fluorescence obtained from cells treated with BP19 alone, to extrapolate the level of mitochondrial labile iron (Mito LI) using an appropriate calibration curve as detailed in Reelfs et al. (2019a). Data were compiled from n = 3–5 measurements per cell line. Mito LI levels are represented as fold change relating to control FEK4 cells (taken as 1). *P < 0.05 Significantly different from healthy cells

**BP19** has been utilised to evaluate the level of mitochondrial labile iron of cultured fibroblasts obtained from Friedreich’s ataxia (FRDA) patients when compared to skin fibroblasts from healthy donors. This study revealed that the mean levels of mitochondrial labile iron in FRDA fibroblasts were on average sixfold higher than those in healthy fibroblasts (Fig. 5) (Reelfs et al. 2019a). The mean levels of mitochondrial labile iron in FRDA cells were 1.11 ± 0.37 μM, whereas those from healthy donors were 0.17 ± 0.12 μM. While it is known that in the neuromuscular disorder FRDA, the decreased iron-sulphur cluster and heme formation by defective frataxin protein causes the pathological accumulation of redox-active labile iron in mitochondrial compartments (Rouault 2016; Llorens et al. 2019; Chiang et al. 2020), our study was the first to provide an estimate of the extent of mitochondrial iron overload in cells derived from FRDA patients.

### Cellular studies with mitochondrial-targeting iron chelators

In an attempt to increase the potency of iron chelation by the use of mitochondria targeted peptides, hexadentate analogues designed in a similar fashion to siderophore structures (Hider and Kong 2010), were investigated (Reelfs et al. 2016). In this context, we synthesized a series of chimeric hexapeptides, containing 3 iron chelating residues attached to a SS-like peptide. The iron-chelating moieties were of either catechol e.g. **BP29** (Fig. 1) or hydroxypyridinone types. Depending on the application, both types of hexapeptide have demonstrated significant potency in various investigations.

### The cytoprotective potential of BP29 against UVA-induced damage in fibroblasts

**Fig. 6:**
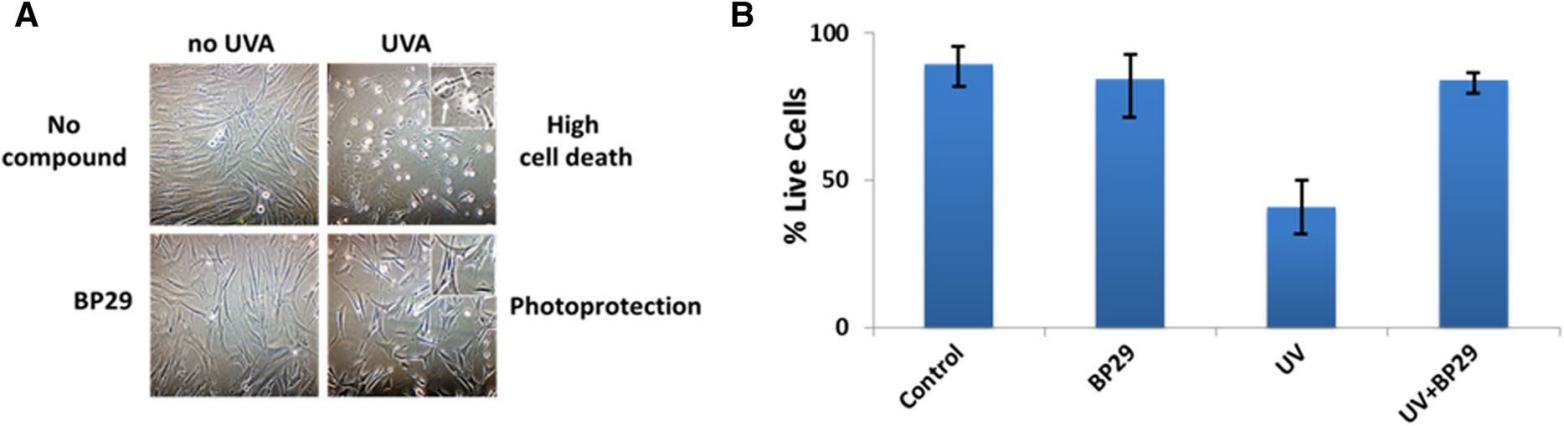
A. Microscopic pictures of cell morphology following BP29 and/or UVA treatment(s). FEK4 fibroblasts were treated as indicated and phase contrast microscopy pictures were taken 24 h post-irradiation. In UVA-irradiated panel (top right), the insert shows cells undergoing cell death following UVA irradiation, with characteristic membrane blebbing. In BP29 + UVA-treated panel (bottom right), the insert highlights the protection afforded by BP29 against UVA irradiation (*i.e.* unaltered cell morphology). **B **Evaluation of the level of cytoprotection afforded by BP29 against UVA-induced cell death. FEK4 cells were first treated (or not) overnight with BP29 at a concentration of 50 μM and then UVA-irradiated or not at a UVA dose of 500 kJ/m^2^. The survival was assessed by flow cytometry using dual staining with AnnexinV and propidium iodide at 24 h post-irradiation. ‘Control’ cells are those that were neither pre-treated with BP29 nor UVA-irradiated. Values are mean ± STD of 3 independent experiments

We reasoned that the use of mitochondria-targeted iron chelators to specifically remove the mitochondrial labile iron may be an effective approach to protect the skin cells against the harmful effects of UVA. In this regard, we demonstrated that pre-treatment of human primary skin fibroblasts with the mitochondria-targeted tri-catechol-based iron chelator linked to mitochondria-homing SS-peptides (**BP29**) exhibits an unprecedented protection against UVA-induced oxidative damage to mitochondrial membrane and the ensuing ATP depletion and necrotic cell death (Fig. 6) (Reelfs et al. 2016). The remarkable potency of this mitochondria-targeted chelator peptide was dependent on its effective uptake by skin cells and its ability to selectively partition to mitochondria, thereby removing the excess of potentially harmful labile iron of the organelle. To our knowledge this is the first mitochondria-targeted iron chelator of its kind to demonstrate promising potential for skin photoprotection against the deleterious effects of UVA component of sunlight.

#### The cytoprotective potential of BP29 against UVA-induced damage in FRDA fibroblasts

**Fig. 7:**
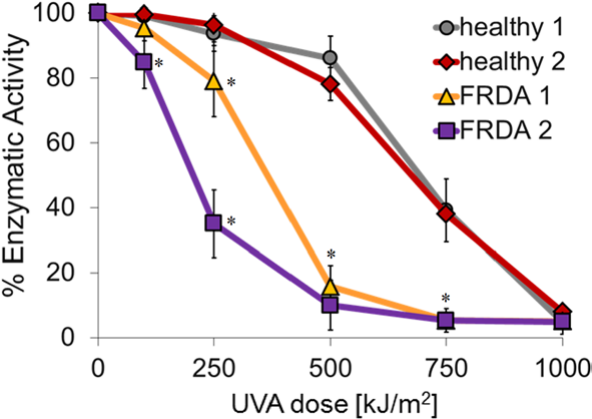
FRDA skin fibroblasts are significantly more sensitive to biologically relevant doses of UVA than their healthy counterparts. Human primary fibroblasts from healthy donors (Healthy 1 and 2) or from donors with FRDA (FRDA 1 and 2) (obtained from Coriell Cell Repositories, New Jersey, USA) were challenged with increasing doses of UVA. Cytotoxicity of the treatment was assessed 24 h post-irradiation by Dimethyl thiazolyl diphenyl tetrazolium bromide (MTT) assay. Values are mean ± SD of 3–5 independent experiments. * *P* < 0.05 Significantly different from healthy (control) cells at corresponding dose

We have recently reported that FRDA skin fibroblasts are highly susceptible to UVA-induced oxidative damage and cell death when compared to healthy control skin fibroblasts (see Reelfs et al. 2019a and Fig. 7). Pre-treatment of FRDA fibroblasts with **BP29** was found to rescue the cells against a high but physiologically-relevant dose of UVA (i.e. 500 kJ/m^2^ equivalent to 3.5 h in sunlight at sea level) (Fig. 7) (Reelfs et al. 2019a). This study highlighted the potential of mitochondria-targeted iron chelators for efficient skin photoprotection in FRDA patients.

#### The cytoprotective potential of BP29 against H_2_O_2_-induced toxicity in FRDA fibroblasts

**Fig. 8:**
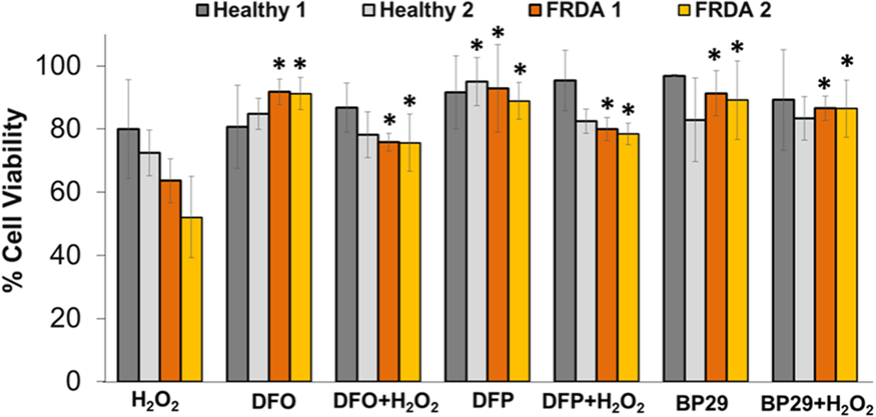
Evaluation of protective effect of chelators against H_2_O_2_-inducted cytotoxicity: human primary fibroblasts from healthy donors (Healthy 1 and 2) or from donors with FRDA (FRDA 1 and 2) (obtained from Coriell Cell Repositories, New Jersey, USA) were pre-treated (or not) overnight with 50 μM desferrioxamine (DFO), deferiprone (DFP) and the mitochondria-targeted **BP29** followed by 1 h treatment with 100 μM H_2_O_2_. The cell viability was assessed 24 h after H_2_O_2_ treatment by MTT assay. Results are shown as mean ± SD of n = 4–8 independent experiments and expressed as the percentage cell viability of untreated control which was set as 100%. *Significantly different from H_2_O_2_ treatment only (P < 0.05)

The higher mitochondrial labile iron of skin fibroblasts from FRDA patients renders them highly susceptible to iron stress and to be appreciably more sensitive to H_2_O_2_-mediated cell death than controls, in line with the importance of the organelles’ labile iron under oxidative stress conditions and pathologies (Wong et al. 1999; Lim et al. 2008; Pourzand et al; 2019). Iron chelation therapy of FRDA patients has demonstrated that chelators such as deferiprone can provide significant and measurable impact on patients’ neurological functions (Boddaert et al. 2007). Nevertheless such chelators lack intracellular organelle specificity. In order to establish whether mitochondrial targeting enhances the efficacy of iron chelators, we compared the effect of **BP29** with two clinically used iron chelators desferrioxamine and deferiprone. Fibroblasts derived from healthy donors and FRDA patients were pre-treated with iron chelators and then challenged with the final H_2_O_2_ concentration of 100 μM (for 1 h). The cytotoxicity tests performed 24 h after the H_2_O_2_ treatment revealed that **BP29** was just as effective as desferrioxamine and deferiprone at preventing oxidative stress in both cell lines (Fig. 8). (Pourzand et al. 2019).

#### The neuroprotective potential of BP29-type molecules against oxidative injuries in Parkinson’s disease

**Fig. 9:**
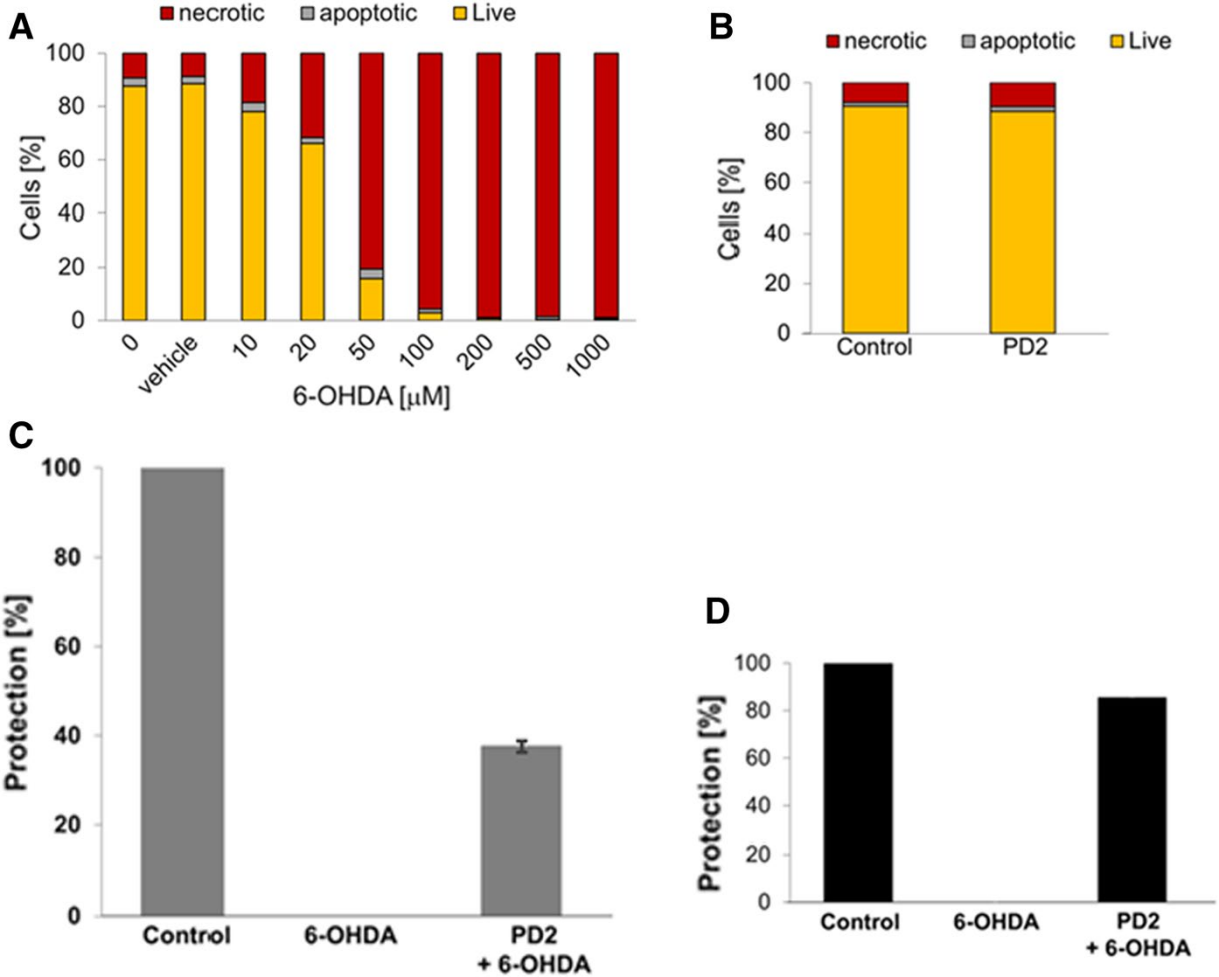
Biological evaluation of the BP29-type mitochondria-targeted iron chelator (PD2) in human neuroblastoma SH-SY5Y cells treated or not with 6-hydroxydopamine (6-OHDA)—**A** *Concentration curve of 6-OHDA cytotoxicity to SHSY5Y cells*. SH-SY5Y cells were challenged overnight with increasing concentrations of 6-OHDA. The percentages of live, apoptotic or necrotic cells were measured by flow cytometry using the dual annexinV-FITC and propidium iodide (PI) assay at 24 h post-treatment time point; double stained cells were considered to be necrotic, whereas Annexin V positive/PI negative cells were apoptotic. Annexin V negative/PI negative were the “Live” cells. Results were obtained from three independent experiments and a representative experiment is depicted here. **B **Evaluation of the cytotoxicity of BP29-type mitochondria-targeted iron chelator in absence of 6-OHDA. Cells were treated (or not) overnight with **PD2** at a final concentration of 50 μM and the percentages of live, apoptotic or necrotic cells were measured by flow cytometry with annexinV-FITC/PI dual staining assay at 24 h post-treatment time point; double stained cells were considered necrotic, whereas Annexin V positive/PI negative cells were apoptotic. Annexin V negative/PI negative were the “Live” cells. Data were obtained from three independent experiments and a representative experiment is depicted here. **C **Evaluation of the protection afforded by BP29-type mitochondria-targeted iron chelator against 6-OHDA-induced cell death. Cells were pre-treated (or not) overnight with **PD2** (50 μM) followed by treatment with 6-OHDA at a final concentration of 50 μM for 18 h. Protection against cell death was measured by flow cytometry with annexinV-FITC/PI dual staining assay; Data were obtained from three independent experiments and expressed as the mean percentage protection (± SD) afforded by **PD2** in cells treated with **PD2** prior to 6-OHD exposure when compared to untreated control (set as 100%). **D **Evaluation of the protection afforded by BP29-type mitochondria-targeted iron chelator against 6-OHDA-induced loss of mitochondrial integrity. Cells were pre-treated (or not) overnight with **PD2** (50 μM) followed by treatment with 6-OHDA at a final concentration of 50 μM for 18 h. Protection against loss of membrane integrity was measured by flow cytometry with the mitochondrial compound tetramethylrhodamine methyl ester TMRM. Data were obtained from three independent experiments and a representative experiment is depicted here

Mitochondrial dysfunction in PD results in detrimental mitochondrial iron overload accompanied with an increase of chelatable redox-active labile iron and consequent excess of labile iron-driven production of harmful reactive oxygen species in specific regions of the nervous system (Devos et al. 2014; Urrutia et al. 2021; Deus et al. 2021). Dopaminergic neuron degeneration follows as a consequence of oxidative stress. This suggests that targeting the organelle’s excess labile iron for removal may be an effective approach for the successful therapy of PD. In order to investigate this possibility, the cytoprotective potential of a **BP29-type** hexadentate mitochondria-targeted iron chelator (**PD2**) was evaluated in SH-SY5Y cells, an in vitro model of PD, against 6-hydroxydopamine (**6-OHDA**)-induced PD-like mitochondrial dysfunction (Fig. 9). Our results demonstrated that the SH-SY5Y cell line, when pre-treated with compound **PD2**, exhibited the highest protection by significantly protecting the cells up to 40% against 6-OHDA-induced necrotic cell death. Our results further revealed that compound **PD2** afforded up to 80% protection against mitochondria membrane depolarization which accompanies 6-OHDA-induced cell death. The cytoprotective properties displayed in vitro by **PD2** demonstrate the validity of such compounds to protect cells against the consequences of mitochondrial dysfunction such as that which are seen in the brain cells from patients with PD (Reelfs et al. 2019b).

## Conclusions

Directing iron-selective chelators to the mitochondria offers a means of chelating mitochondrial excess iron with minimal iron scavenging activity in the cytosol, nucleus and lysosome. In principle this should reduce the toxicity of such chelators. In this study we describe the synthesis of one such molecule. We have demonstrated that such chelators, modelled on siderophore structure, have cytoprotective potential in a range of cell types.

It will be important to be able to measure the labile iron levels in mitochondria and to this end we report the synthesis and design of a mitochondrial targeted iron selective fluorescent probe. Using this probe, we have demonstrated a large increase in the mitochondrial labile iron pool of fibroblasts isolated from FRDA patients.

### Synthetic details

Materials and chemicals were purchased from Acros Organics, Merck, Fisher Scientific International, and Fluorochem Ltd and were reagent grade or better. Solvents and NMR solvents were purchased from Fisher Scientific, Merck KGaA, and VWR. Silica gel for column chromatography was purchased from Merck. All samples were dried in a vacuum oven connected to a vacuum pump (BOC-Edwards.). Silica gel 60 F254, Merck pre-coated aluminum sheets were employed for thin-layer chromatography (TLC) and spots were visualized under UV light. ^1^H NMR and ^13^C NMR spectra were recorded on A Bruker Avance III HD NanoBay 400 MHz NMR with a 5 mm ^1^H/^13^C/^15^N/^31^P QNP probe equipped with z-gradient. Tetramethylsilane (TMS) was used in all NMR experiments as internal standard and chemical shift (*δ*) values are given in ppm. Low resolution mass spectra were obtained on a Thermofisher LCQ DECA XP ion trap mass spectrometer or a Waters—Micromass ZQ—Single quadrupole mass spectrometer. High-resolution Mass Spectrometry (HRMS) was conducted on a Thermo Fisher Scientific Exactive Mass Spectrometer operating in positive electrospray ionisation mode.

**3** (2,5-dioxopyrrolidin-1-yl (*tert*-butoxycarbonyl)-l-phenylalaninate). *N*, *N*′-Dicyclohexylcarbodiimide (DCC) (1.01 g, 4.90 mmol, 1.3 eq) and *N*-hydroxysuccinimide (0.56 g, 4.90 mmol, 1.3 eq) were added to a solution of Boc-l-Phe-OH (**2**) (1.00 g, 3.76 mmol, 1.0 eq) dissolved in 50 mL of dimethylformamide (DMF) and stirred for 3 h at room temperature. The mixture was filtered, the filtrate was concentrated under reduced pressure and the obtained compound **3** was used without any further purification. Yield 99%.

**5** (*N*^2^-((*tert*-butoxycarbonyl)-l-phenylalanyl)-*N*^w^-((2,2,4,6,7-pentamethyl-2,3-dihydrobenzofuran-5-yl)sulfonyl)-d-arginine). Compound **3** (0.93 g, 2.57 mmol, 1.1 eq) was added to a solution of d-Arg(Pbf)-OH (**4**) (1.00 g, 2.34 mmol, 1.0 eq) and triethylamine (1.30 mL, 9.37 mmol, 4.0 eq) in DMF (50 mL). The reaction was stirred at room temperature for 24 h. The solvent was removed under reduced pressure and the crude product was purified by column chromatography using a gradient of EtOH/NH_4_OH/DCM/Pet. Et. from13.7:1.7:71.9:12.7 to 23.5:4.7:62.5:9.4 as eluents. Yield 75%. ^1^H NMR (400 MHz, Methanol-*d*_4_) δ 7.22 (d, *J* = 6.1 Hz, 5H), 7.19–7.11 (m, 1H), 4.29 (d, *J* = 6.1 Hz, 1H), 4.23 (s, 1H), 3.11 (dd, *J* = 13.7, 5.5 Hz, 3H), 2.99 (s, 2H), 2.82 (dd, *J* = 13.7, 8.8 Hz, 1H), 2.58 (s, 3H), 2.52 (s, 3H), 2.08 (s, 3H), 1.75 (s, 1H), 1.56 (dt, *J* = 13.7, 7.5 Hz, 1H), 1.45–1.29 (m, 15H). ^13^C NMR (101 MHz, Methanol-*d*_4_) δ 173.63, 159.84, 157.52, 139.40, 138.65, 134.39, 133.49, 130.45, 130.35, 129.47, 129.26, 127.72, 127.52, 126.02, 118.44, 87.64, 80.70, 57.77, 54.45, 43.97, 39.39, 30.56, 28.74, 28.70, 28.66, 26.44, 19.65, 18.43, 12.56. HRMS Calculated [M + H]: 674.3218; Found [M + H]; 674.3208.

**8** ((*S*)-5-(3-(benzyloxy)-2-methyl-4-oxopyridin-1(4*H*)-yl)-2-((*tert*-butoxycarbonyl)amino)pentanoic acid). 3-(Benzyloxy)-2-methyl-4*H*-pyran-4-one (**7**) (1.1 g, 4.64 mmol, 1.1 eq) was added to a stirred solution of Boc-l-Orn-OH (**6**) (980 mg, 4.22 mmol, 1.0 eq) in ethanol/water 1:1 (20 mL) and the pH was adjusted to 10.5 using 1 N sodium hydroxide solution and the mixture refluxed for 24 h. The solvent was evaporated under vacuum. The reaction was acidified with 6 N HCl until pH 7 and extracted with DCM (3 × 40 mL). The organic layers were dried over anhydrous sodium sulfate, filtered, and rotary evaporated to give an orange oil. Further purification was obtained by flash column chromatography using a gradient of DCM/MeOH from 8:2 to 6:4 as eluents. Yield 32%. ^1^H NMR (400 MHz, Methanol-*d*_4_) δ 7.71 (d, *J* = 7.4 Hz, 1H), 7.35 (ddd, *J* = 11.3, 6.4, 3.2 Hz, 5H), 6.49 (d, *J* = 7.4 Hz, 1H), 5.07 (s, 2H), 4.00 (tt, *J* = 14.2, 6.4 Hz, 3H), 2.15 (s, 3H), 1.89–1.54 (m, 4H), 1.44 (s, 9H). ^13^C NMR (101 MHz, Methanol-*d*_4_) δ 174.58, 158.07, 145.28, 141.24, 138.35, 130.35, 129.46, 117.34, 80.52, 74.57, 54.80, 29.99, 28.78, 28.09, 12.85. HRMS Calculated [M + H]: 431.2711; Found [M + H]; 431.2171.

**10** (benzyl *tert*-butyl (3-amino-3-oxopropane-1,2-diyl)(*S*)-dicarbamate). To Z-l-Dap(Boc)-OH (**9**) (1.00 g, 2.95 mmol, 1.0 eq) in dry acetonitrile (40 ml), Boc_2_O (1.29 g, 5.91 mmol, 2.0 eq), ammonium bicarbonate (467 mg, 5.91 mmol, 2.0 eq) and pyridine (0.88 mL, 10.91 mmol, 3.7 eq) were added, and the reaction mixture was stirred for 16 h at rt. To the product mixture, water (40 mL) was added and the volume was reduced under reduced pressure to 40 mL. The solid product was filtered, washed with water (4 × 50 mL) and hexane (4 × 50 mL), and dried in vacuo to give **10**. Yield 96%. ^1^H NMR (400 MHz, Methanol-*d*_4_) δ 8.21 (d, *J* = 4.4 Hz, 6H), 8.04–7.84 (m, 2H), 7.59 (s, 1H), 5.87 (d, *J* = 5.7 Hz, 2H), 4.86 (q, *J* = 7.5 Hz, 1H), 2.21 (s, 9H). HRMS Calculated [M + H]: 338.1710; Found [M + H]; 338.1361.

**11** (benzyl (*S*)-(1,3-diamino-1-oxopropan-2-yl)carbamate). Compound **10** (1.00 g, 2.96 mmol) was solubilized in 10 ml of dichloromethane, then 10 ml of trifluoroacetic acid were added and the reaction was stirred for 1 h. The volume of the mixture was reduced under reduced pressure to 5 mL then cold diethyl ether (50 mL) was added. The suspension was centrifuged, and the precipitate (**11**) was further washed with diethyl ether (2 × 50 mL). Molecule **11** was then dried under vacuum and used without further purification. Yield 99% ^1^H NMR (400 MHz, Methanol-*d*_4_) δ 7.02 (bs, 1H), 8.35–8.21 (m, 6H), 3.86 (s, 2H), 2.21 (dd, *J* = 13.1, 5.3 Hz, 1H), 1.98 (dd, *J* = 13.1, 8.0 Hz, 1H).

**13** (benzyl (*S*)-(1-amino-3-((5-(dimethylamino)naphthalene)-1-sulfonamido)-1-oxopropan-2-yl)carbamate). To a stirred solution of molecule **11** (1.0 g, 4.20 mmol, 1 eq) and triethylamine (0.58 mL, 4.20 mmol, 1 eq) in dry dichloromethane (20 mL) at room temperature, a solution of dansyl chloride (**12**) (1,36 g, 5.04 mmol 1.2 eq) in dry dichloromethane (10 mL) was added during 5 min and stirring of the reaction mixture was continued for 24 h. The reaction mixture was washed with water, the solvent was removed under vacuum and the residue was column chromatographed using a gradient of DCM/MeOH from 95:5 to 80:20 as eluents. Yield 70%. ^1^H NMR (400 MHz, Chloroform-*d*/Methanol-*d*_4_) δ 8.50–8.53 (m, 1H), 8.35–8.21 (m, 1H), 8.16 (d, *J* = 7.5 Hz, 1H), 7.57–7.41 (m, 2H), 7.29 (d, *J* = 4.8 Hz, 5H), 7.17 (d, *J* = 7.5 Hz, 1H), 5.00 (d, *J* = 3.3 Hz, 2H), 4.18 (t, *J* = 5.8 Hz, 1H), 3.18 (dt, *J* = 13.1, 6.3 Hz, 2H), 2.84 (s, 6H). ^13^C NMR (101 MHz, Chloroform-*d*/Methanol-*d*_4_) δ 173.90, 157.38, 152.52, 136.86, 135.39, 131.07, 130.58, 130.15, 129.84, 129.01, 128.88, 128.66, 128.42, 123.78, 119.51, 115.94, 67.59, 55.19, 45.69, 44.68. HRMS Calculated [M + H]: 471.1697; Found [M + H]; 471.1689.

**14** ((*S*)-2-amino-3-((5-(dimethylamino)naphthalene)-1-sulfonamido)propanamide). To a solution of compound **13** (1.0 g, 2.12 mmol) in THF/MeOH 1:1 (20 mL), was added 10% palladium on carbon (150 mg), and the mixture was stirred under an atmosphere of H_2_ for 12 h. The suspension was filtered through a pad of celite and the filtrate was concentrated under reduced pressure giving a yellowish oil (**14**). Molecule **14** was then dried under vacuum and used without further purification. Yield 99%. ^1^H NMR (400 MHz, Chloroform-*d*/Methanol-*d*_4_) δ 8.56 (d, *J* = 8.5 Hz, 1H), 8.34 (d, *J* = 8.5 Hz, 1H), 8.30–8.17 (m, 1H), 7.65–7.46 (m, 2H), 7.22 (d, *J* = 7.5 Hz, 1H), 4.13 (d, *J* = 8.5 Hz, 1H), 3.34 (s, 2H), 2.90 (s, 6H). ^13^C NMR (101 MHz, Chloroform-*d*/Methanol-*d*_4_) δ 171.07, 152.26, 134.49, 131.05, 130.25, 129.78, 129.68, 128.92, 123.58, 119.18, 115.83, 53.97, 45.59, 44.49. HRMS Calculated [M + H]: 337.1329; Found [M + H]; 337.1325.

**15** (*tert*-butyl ((*S*)-1-(((*S*)-1-amino-3-((5-(dimethylamino)naphthalene)-1-sulfonamido)-1-oxopropan-2-yl)amino)-5-(3-(benzyloxy)-2-methyl-4-oxopyridin-1(4*H*)-yl)-1-oxopentan-2-yl)carbamate). To a stirred solution of compound **8** (1.0 g, 2.32 mmol, 1.0 eq) and hydroxybenzotriazole (HOBt) (376 mg, 2.78 mmol, 1.2 eq) in DMF (50 ml) was added 1-Ethyl-3-(3-dimethylaminopropyl)carbodiimide (EDC) (432 mg, 2.78 mmol, 1.2 eq) at 0 °C. The mixture was stirred at this temperature for 5 min. Molecule **14** (780 mg, 2.32 mmol, 1.0 eq) and triethylamine (0.308 mL, 2.32 mmol, 1.0 eq) were then added to the solution. The resulting mixture was stirred at room temperature for 24 h. After evaporation of the solvent, the crude mixture was washed with H_2_O and extracted with DCM. The organic phase was dried over sodium sulfate and dried. The residue was column chromatographed on silica gel using a gradient of DCM/MeOH from 96:4 to 90:10 as eluents. Yield 90%. ^1^H NMR (400 MHz, Methanol-*d*_4_) δ 8.52 (dt, *J* = 8.5, 1.1 Hz, 1H), 8.31 (d, *J* = 8.5 Hz, 1H), 8.14 (dd, *J* = 7.4, 1.1 Hz, 1H), 7.65 (d, *J* = 7.4 Hz, 1H), 7.54 (ddd, *J* = 8.5, 7.4, 6.0 Hz, 2H), 7.35 (dd, *J* = 5.1, 2.0 Hz, 2H), 7.29 (dd, *J* = 5.1, 2.0 Hz, 2H), 7.24–7.20 (m, 1H), 6.46 (d, *J* = 7.4 Hz, 1H), 5.03 (s, 2H), 4.41 (t, *J* = 5.5 Hz, 1H), 4.08–3.77 (m, 3H), 3.21 (dd, *J* = 5.6, 3.4 Hz, 2H), 2.83 (s, 6H), 2.16 (s, 3H), 1.90–1.54 (m, 4H), 1.46 (s, 9H). ^13^C NMR (101 MHz, Methanol-*d*_4_) δ 173.21, 172.66, 157.04, 151.86, 145.66, 143.72, 139.77, 137.06, 135.06, 130.05, 129.84, 129.43, 128.81, 128.70, 128.03, 127.98, 127.93, 122.97, 118.91, 116.06, 115.15, 79.92, 73.15, 54.97, 53.35, 53.14, 44.43, 43.34, 27.85, 27.44, 26.51, 11.52. HRMS Calculated [M + H]: 749.3327; Found [M + H]; 749.3318.

**16** ((*S*)-2-amino-*N*-((*S*)-1-amino-3-((5-(dimethylamino)naphthalene)-1-sulfonamido)-1-oxopropan-2-yl)-5-(3-(benzyloxy)-2-methyl-4-oxopyridin-1(4*H*)-yl)pentanamide). Compound **15** (1.00 g, 1.33 mmol) was solubilized in 10 mL of dichloromethane, then 10 mL of trifluoroacetic acid were added and the reaction was stirred for 1 h. The volume of the mixture was reduced under reduced pressure to 5 mL then cold diethyl ether (50 mL) was added. The suspension was centrifuged, and the precipitate (**16**) was further washed with diethyl ether (2 × 50 mL). Molecule **16** was then dried under vacuum and used without further purification. Yield 99%. ^1^H NMR (400 MHz, Methanol-*d*_4_) δ 8.67 (d, *J* = 8.4 Hz, 1H), 8.55 (d, *J* = 8.4 Hz, 1H), 8.32 (dd, *J* = 16.0, 7.2 Hz, 2H), 7.88–7.62 (m, 3H), 7.38 (dd, *J* = 22.4, 5.2 Hz, 5H), 7.23 (d, *J* = 7.2 Hz, 1H), 4.48 (s, 1H), 4.37 (d, *J* = 7.2 Hz, 2H), 4.05 (s, 1H), 3.24 (d, *J* = 9.5 Hz, 8H), 2.52 (s, 3H), 2.00 (q, *J* = 6.1, 5.6 Hz, 4H). ^13^C NMR (101 MHz, Methanol-*d*_4_) δ 173.26, 169.66, 165.86, 151.15, 145.04, 142.99, 137.29, 136.84, 130.68, 130.62, 129.99, 129.83, 129.81, 129.66, 129.59, 129.22, 125.63, 123.14, 118.04, 117.42, 114.60, 114.23, 76.20, 56.87, 55.00, 53.72, 46.45, 46.44, 44.67, 28.89, 26.34, 13.64. HRMS Calculated [M + H]: 649.2803; Found [M + H]; 649.2801.

**17** (*tert*-butyl ((*S*)-1-(((*R*)-1-(((*S*)-1-(((*S*)-1-amino-3-((5-(dimethylamino)naphthalene)-1-sulfonamido)-1-oxopropan-2-yl)amino)-5-(3-(benzyloxy)-2-methyl-4-oxopyridin-1(4*H*)-yl)-1-oxopentan-2-yl)amino)-1-oxo-5-(3-((2,2,4,6,7-pentamethyl-2,3-dihydrobenzofuran-5-yl)sulfonyl)guanidino)pentan-2-yl)amino)-1-oxo-3-phenylpropan-2-yl)carbamate). To a stirred solution of compound **5** (1.0 g, 1.48 mmol, 1.0 eq) and hydroxybenzotriazole (HOBt) (240 mg, 1.78 mmol, 1.2 eq) in DMF (50 mL) was added 1-Ethyl-3-(3-dimethylaminopropyl)carbodiimide (EDC) (276 mg, 1.78 mmol, 1.2 eq) at 0 °C. The mixture was stirred at this temperature for 5 min. Molecule **16** (962 mg, 1.48 mmol, 1.0 eq) and triethylamine (0.206 mL, 1.48 mmol, 1.0 eq) were then added to the solution. The resulting mixture was stirred at room temperature for 24 h. After evaporation of the solvent, the crude mixture was washed with H2O and extracted with DCM. The organic phase was dried over sodium sulfate and dried. The residue was column chromatographed on silica gel using EtOH/NH_4_OH/DCM/Pet. Et. 13.7:1.7:71.9:12.7 as eluent. Yield 90%. ^1^H NMR (400 MHz, Methanol-*d*_4_) δ 8.53 (dd, *J* = 8.7, 5.3 Hz, 1H), 8.31 (d, *J* = 8.7 Hz, 1H), 8.20–8.09 (m, 1H), 7.68 (t, *J* = 7.0 Hz, 1H), 7.58–7.46 (m, 2H), 7.38–7.33 (m, 2H), 7.29 (tt, *J* = 4.8, 2.7 Hz, 3H), 7.25–7.08 (m, 6H), 6.50–6.41 (m, 1H), 5.06–4.96 (m, 2H), 4.42 (dt, *J* = 10.1, 3.7 Hz, 2H), 4.31 (td, *J* = 10.1, 9.6, 6.9 Hz, 2H), 3.94 (t, *J* = 6.9 Hz, 2H), 3.30–3.04 (m, 4H), 2.84 (s, 10H), 2.49–2.58 (m, 5H), 2.19 (d, *J* = 2.1 Hz, 3H), 2.12–1.41 (m, 11H), 1.45–1.17 (m, 16H). ^13^C NMR (101 MHz, Methanol-*d*_4_) δ 173.32, 173.16, 172.52, 172.33, 172.12, 171.97, 158.46, 156.79, 151.86, 145.74, 143.66, 139.83, 138.04, 137.08, 135.02, 132.94, 132.17, 130.05, 129.82, 129.42, 129.01, 128.76, 128.73, 128.13, 128.01, 127.89, 126.30, 124.72, 124.64, 122.99, 118.90, 117.05, 116.11, 115.15, 86.30, 86.26, 79.40, 73.13, 55.91, 53.66, 53.29, 53.14, 52.92, 44.42, 44.40, 43.53, 42.56, 27.67, 27.33, 26.57, 18.42, 18.36, 17.18, 11.60, 11.56, 11.52, 11.22, 11.19. HRMS Calculated [M + H]: 1304.5842; Found [M + H]; 1304.5851.

**1 (BP19)** ((*S*)-*N*-((*S*)-1-amino-3-((5-(dimethylamino)naphthalene)-1-sulfonamido)-1-oxopropan-2-yl)-2-((*R*)-2-((*S*)-2-amino-3-phenylpropanamido)-5-guanidinopentanamido)-5-(3-hydroxy-2-methyl-4-oxopyridin-1(4*H*)-yl)pentanamide). Compound **17** (1.00 g, 0.76 mmol) was solubilized in 20 ml of trifluoroacetic acid and the reaction was stirred for 24 h. The volume of the mixture was reduced under reduced pressure to 5 mL then cold diethyl ether (50 mL) was added. The suspension was centrifuged, and the precipitate (**1**) was further washed with diethyl ether (2 × 50 mL). Molecule **1** was then dried under vacuum and used without further purification for biophysical studies. Samples used in biological assays were further purified by preparative HPLC. Yield 99%. ^1^H NMR (400 MHz, Methanol-*d*_4_/D_2_O/CD_3_COCD_3_) δ 8.54 (d, *J* = 8.5 Hz, 1H), 8.35 (q, *J* = 8.5, 7.8 Hz, 1H), 8.17 (d, *J* = 7.8 Hz, 1H), 8.04 (s, 1H), 7.64 (p, *J* = 8.5, 7.8 Hz, 2H), 7.43–7.16 (m, 6H), 7.03 (s, 1H), 4.55–4.14 (m, 6H), 3.30–2.82 (m, 12H), 2.58 (s, 3H), 1.93 (s, 6H), 1.66 (s, 2H). ^13^C NMR (101 MHz, Chloroform-*d*/Methanol-*d*_4_) δ 172.89, 172.60, 169.26, 168.84, 161.91, 156.93, 134.78, 133.85, 129.73, 129.59, 129.41, 129.34, 129.00, 128.94, 128.37, 128.32, 127.66, 127.61, 123.95, 120.19, 116.20, 55.51, 54.06, 53.40, 45.22, 43.42, 40.60, 36.89, 30.14, 28.51, 27.68, 26.51, 24.49, 11.89. HRMS Calculated [M + H]: 862.4029; Found [M + H]; 862.4016.
